# Targeting USP14/UCHL5: A Breakthrough Approach to Overcoming Treatment-Resistant FLT3-ITD-Positive AML

**DOI:** 10.3390/ijms251910372

**Published:** 2024-09-26

**Authors:** Ayako Nogami, Hideki Jose Amemiya, Hiroki Fujiwara, Yoshihiro Umezawa, Shuji Tohda, Toshikage Nagao

**Affiliations:** 1 Department of Laboratory Medicine, Graduate School of Medical and Dental Sciences, Tokyo Medical and Dental University (TMDU), 1-5-45 Yushima, Bunkyoku, Tokyo 113-8510, Japan; 2 Department of Hematology, Graduate School of Medical and Dental Sciences, Tokyo Medical and Dental University (TMDU), Tokyo 113-8510, Japan

**Keywords:** USP14, UCHL5, FLT3-ITD-positive AML, treatment resistance, deubiquitination

## Abstract

FMS-like tyrosine kinase 3 (FLT3) internal tandem duplication (ITD) mutations in acute myeloid leukemia (AML) are associated with poor prognosis and therapy resistance. This study aimed to demonstrate that inhibiting the deubiquitinating enzymes ubiquitin-specific peptidase 14 (USP14) and ubiquitin C-terminal hydrolase L5 (UCHL5) (USP14/UCHL5) with b-AP15 or the organogold compound auranofin (AUR) induces apoptosis in the ITD-transformed human leukemia cell line MV4-11 and mononuclear leukocytes derived from patients with FLT3-ITD-positive AML. This study included patients diagnosed with AML at Tokyo Medical and Dental University Hospital between January 2018 and July 2024. Both treatments blocked downstream FLT3 pathway events, with the effects potentiated by USP14 knockdown. Both treatments inhibited FLT3 deubiquitination via K48 and disrupted translation initiation via 4EBP1, a downstream FLT3 target. FLT3 was downregulated in the leukemic cells, with the associated activation of stress-related MAP kinase pathways and increased NF-E2-related factor 2. Furthermore, the overexpression of B-cell lymphoma-extra-large and myeloid cell leukemia-1 prevented the cell death caused by b-AP15 and AUR. These results suggest that inhibiting USP14/UCHL5, which involves multiple regulatory mechanisms, is a promising target for novel therapies for treatment-resistant FLT3-ITD-positive AML.

## 1. Introduction

FMS-like tyrosine kinase 3 (FLT3), a tyrosine kinase-type receptor expressed in hematopoietic progenitor cells [1,2], harbors internal tandem duplication (ITD) mutations (FLT3-ITDs) in its transmembrane domain, which are the most prevalent mutations occurring in approximately 20–30% of patients with acute myeloid leukemia (AML). These mutations are strongly associated with a poor prognosis [3,4,5]. Despite the development of FLT3-targeted tyrosine kinase inhibitors, single agent therapies have proven insufficient [1]. Previously, we demonstrated that the molecular basis of FLT3-ITD-induced resistance to treatment is mediated by the mTOR/4EBP1/Mcl-1 pathway via STAT5 activation. This can be overcome by PI3K/AKT pathway inhibitors or combined with proteasome inhibitors [6,7]. The ubiquitin–proteasome system (UPS) is crucial for regulating post-translational protein activity and stability in normal and tumor cells. This is achieved through cell cycle control, signal transduction, and transcriptional regulation [8]. In the UPS, E3 ligases (RING, UBOX, RBR, or HECT types) catalyze ubiquitin transfers, whereas deubiquitinating enzymes (DUBs) detach the ubiquitin chain from the substrate [9]. Examples of DUBs include ubiquitin-specific protease (USP), ubiquitin C-terminal hydrolase (UCH), ovarian tumor ubiquitin, Josephin domain, and Jab1/Mov34/Mpr1/Pad1 [10,11,12]. Among them, ubiquitin-specific peptidase 14 (USP14) and ubiquitin C-terminal hydrolase L5 (UCHL5) bind reversibly to 19S regulatory particles and act on Rpn11 and Rpn13, respectively, affecting the incorporation of intracellular proteins into the proteasome [13]. Recently, the small molecule inhibitors of USP14 and UCHL5 (USP14/UCHL5) [14,15,16], such as b-AP15 and auranofin (AUR), have been reported to suppress their deubiquitination activity [15,17,18]. This results in the apoptosis of cancer cells, regardless of TP53 or BCL2 levels [14]. Moreover, b-AP15 has demonstrated activity in various hematological cancers, including multiple myeloma [19], Waldenström’s macroglobulinemia [20], and diffuse large B-cell lymphoma [21], making it a novel therapeutic target. In contrast, AUR, a gold-based compound, has been used for treating rheumatoid arthritis since 1985 [22]. The primary mechanism of action involves the inhibition of thioredoxin-disulfide reductase (TrxR) [23,24,25]. Other mechanisms of action have been reported, and those involving direct effects on I kappa B kinase [26,27], protein kinase C iota [28,29], and forkhead box O3 [30] have been confirmed. Furthermore, AUR exhibits inhibitory activity against cellular growth through various mechanisms, including the inhibition of the Janus kinase (JAK) 2/signal transducers and activators of transcription (STAT) 3 pathway in myeloma cells [31] and the phospho-inositide 3-kinase (PI3K)/protein kinase B (Akt)/mechanistic target of the rapamycin (mTOR) pathway in non-small cell lung cancer cells [32]. Our previous research focused on the regulatory role of UPS in FLT3-ITD AML; we found that FLT3-ITD misfolding leads to FLT3 degradation via Cbl-b and c-Cbl in the UPS [33]. Furthermore, we reported that USP9X inhibits oxidative stress, with its dysfunction leading to the degradation of large aggregates via a compensatory mechanism in aggresomes [34]. These findings suggest that AML cells employ diverse regulatory mechanisms for FLT3-ITD. However, the appropriate decomposition control of ITD by other DUBs, particularly USP14 and UCHL5, remains unexplored. In addition, reports on the effects of their inhibition on FLT3-ITD-positive AML and the interaction between USP14 and ITD are limited. Therefore, this study aimed to investigate FLT3-ITD regulation by DUBs by demonstrating that the inhibition of the deubiquitinating enzymes, USP14/UCHL5, by the specific inhibitor, b-AP15, and nonspecific inhibitor, AUR, effectively induces apoptosis in FLT3-ITD-transformed cells. The impact of their inhibition on FLT3-ITD downregulation and oxidative stress, as well as on NF-E2-related factor 2 (Nrf2), was also investigated.

## 2. Results

### 2.1. b-AP15 and AUR Potently Induce Apoptosis in ITD-Positive Leukemia Cell Lines via a Mechanism That Can Be Inhibited by Antioxidants

**Figure 1:**
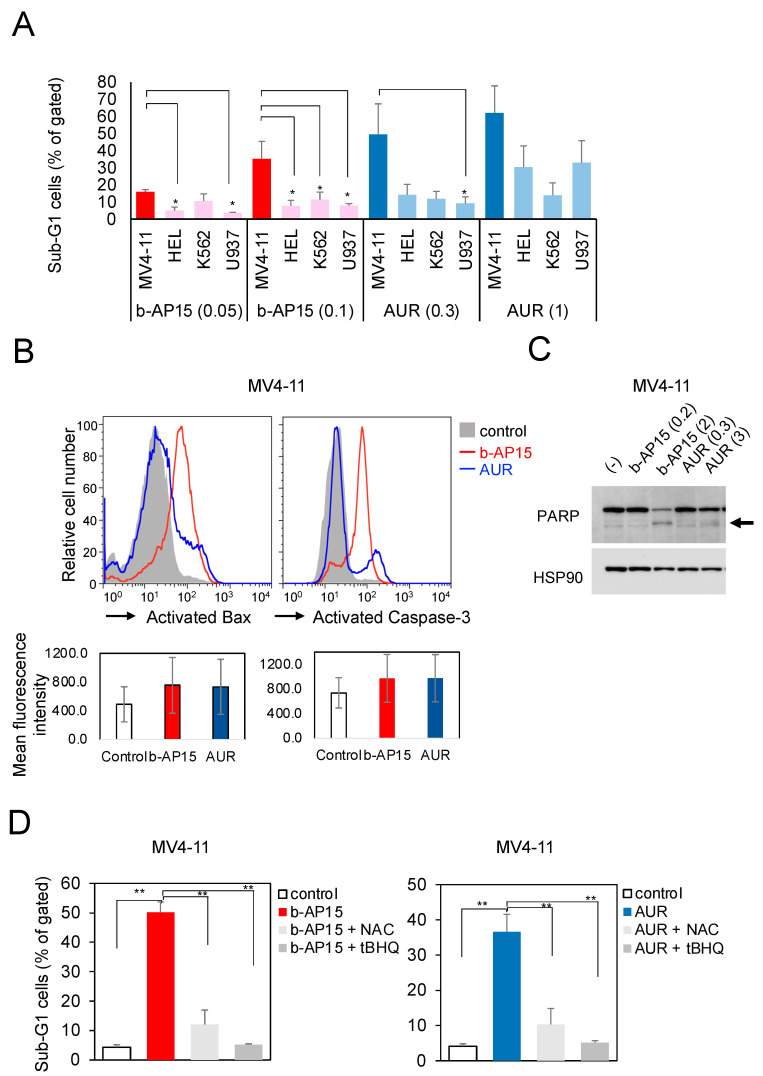
b-AP15 and auranofin (AUR) potently induce apoptosis in ITD-positive leukemia cell lines via a mechanism that can be inhibited by antioxidants. (**A**) The MV4-11, HEL, K562, or U937 cells were cultured for 20 h in the absence (control) or presence of 0.05 or 0.1 µM of b-AP15 or 0.3 or 1 µM of AUR. The cellular DNA content was analyzed by flow cytometry, and the percentage of apoptotic cells with sub-G1 DNA content was identified. Similar experiments were repeated three times. The columns indicate the means, and the error bars indicate the standard errors. The differences in other cell groups relative to the MV4-11 group were tested using Dunnett’s multiple comparison method, with *p* < 0.05 indicated by *. Abbreviation: ITD, internal tandem duplication. (**B**) The MV4-11 cells were cultured in the absence (control) or presence of 0.3 µM of b-AP15 or 0.3 µM of AUR for 5 h. The expression of activated Bax and cleaved caspase-3 was analyzed by flow cytometry. The horizontal axis represents the fluorescence intensity, whereas the vertical axis depicts the relative number of cells exhibiting that fluorescence intensity when the cell count mode is 100. Abbreviation: Bax, Bcl-2-associated X protein. (**C**) The MV4-11 cells were cultured for 3 h in the absence (control) or presence of 0.2 or 2 µM of b-AP15 or 0.3 or 3 µM of AUR. Subsequently, the cells were lysed and subjected to an immunoblot analysis using antibodies directed against the specified proteins. The loading control was heat shock protein 90 (HSP90). Abbreviation: PARP, poly (ADP-ribose) polymerases. The arrow indicates the level of cleaved PARP. (**D**) The MV4-11 cells were cultured in the absence (control) or presence of 0.3 µM of b-AP15 or 0.3 µM of AUR for 6 h. In the presence of b-AP15 or AUR, the cultures were grown with or without 1 mM of N-acetyl-L-cysteine (NAC) or 10 µM of tertiary butylhydroquinone (tBHQ). The cellular DNA content was analyzed by flow cytometry, and the percentage of apoptotic cells with sub-G1 DNA content was indicated. Similar experiments were conducted four times, with the columns representing the means and the error bars representing the standard errors. The Bonferroni multiple comparison test was employed to assess the differences between the other cell groups and the b-AP alone or AUR alone treatment groups, with *p* < 0.01 indicated as **.

First, we investigated the effects of b-AP15, a specific inhibitor of USP14/UCHL5, and AUR, a gold compound with similar inhibitory activity, on the cell cycle of leukemia cell lines. Both compounds induced concentration-dependent apoptosis in FLT3-ITD-positive MV4-11 cells, as shown by the sub-G1 phase in the cell cycle, with lesser effects in other leukemia cell lines (Figure 1A). The IC50 values of both drugs in MV4-11 cells, determined in other experiments, were 0.23 and 0.50 μM, respectively. Subsequently, we examined the activation of Bcl-2-associated X protein (Bax) and caspase-3 to understand the mechanism of apoptosis induction in MV4-11 cells, and the results showed that both b-AP15 and AUR activated Bax and caspase-3 at the same concentrations, inducing their apoptosis (Figure 1B). Moreover, a Western blotting analysis revealed that both compounds induced poly (ADP-ribose) polymerase (PARP) cleavage (Figure 1C). In addition, the impact of reactive oxygen species (ROS) regulation by USP was investigated. The pro-apoptotic effects of the b-AP15 and AUR treatments were antagonized by the antioxidants tertiary butylhydroquinone and N-acetylcysteine (NAC) (Figure 1D). To ascertain the potential for direct involvement, we evaluated the ROS production in the MV4-11 cells following the b-AP15 or AUR treatment. The findings showed that the ROS production was relatively weak (Figure S1). These results indicate that inhibiting USP14/UCHL5 strongly induces apoptosis via intrinsic pathways in ITD-positive leukemia cells, and this effect is inhibited by antioxidants.

### 2.2. The Increased Ubiquitin (Ub) Protein and the Activation of Stress-Related MAP Kinase Pathways Produced by USP14/UCHL5 Inhibition, Unlike Those Produced by Bortezomib, Are Circumvented after Antioxidant Administration

**Figure 2:**
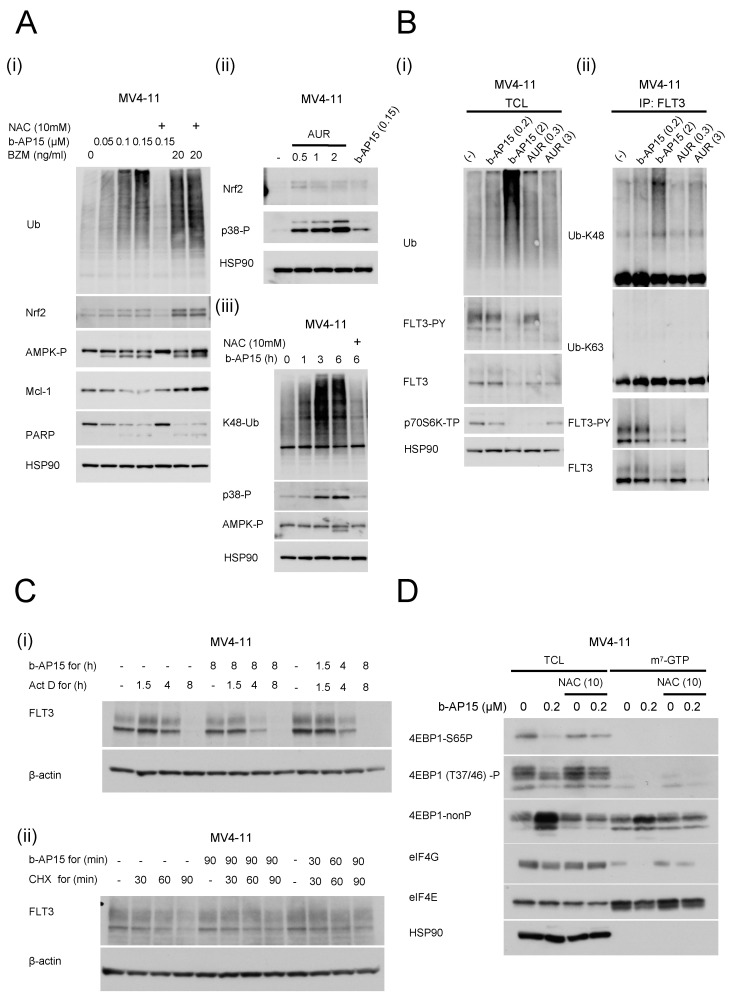
The increased Ub protein and the activation of stress-related MAP kinase pathways produced by USP14/UCHL5 inhibition are mitigated by antioxidant administration, unlike with the bortezomib treatment. (**A**) (**i**) The MV4-11 cells were cultured for 5 h in the absence (control) or presence of 0.05, 0.1, or 0.15 µM of b-AP15 or 20 ng/mL of bortezomib (BZM). The b-AP15 or BZM were added in the presence or absence of 1 mM of NAC. Subsequently, the cells were lysed and subjected to an immunoblot analysis using the specified antibodies. HSP90 served as the loading control. Abbreviations: ub, ubiquitin; Nrf-2, NF-E2-related factor 2; AMPK-P, phosphor-AMPK; and HSP90, heat shock protein 90. (**ii**) The cells were cultured in the absence (control) or presence of 0.15 µM of b-AP15 or 0.5, 1, or 2 µM of AUR for 5 h; then lysed and subjected to an immunoblot analysis using the indicated antibodies. HSP90 served as the loading control. (**iii**) The MV4-11 cells were cultured in the absence (control) or presence of 0.1 µM of b-AP15 for the times indicated in the figure. The b-AP15 was cultured with or without 1 mM of NAC. Subsequently, the cells were lysed and subjected to an immunoblot analysis using the antibodies directed against the specified proteins. HSP90 was utilized as the loading control. Abbreviations: K48-Ub, K48-linkage specific polyubiquitin; and p38-P, phospho-p38. (**B**) (**i**) The MV4-11 cells were cultured in the absence (control) or presence of 0.2 or 2 µM of b-AP15 or 0.3 or 3 µM of AUR for 5 h. Whole-cell lysates (TCLs) and (**ii**) immunoprecipitates (IPs) with anti-FLT3 antibodies were subjected to immunoblotting using the indicated antibodies. Abbreviations: FLT3-PY, phospho-FMS-like tyrosine kinase 3; p70S6K-TP, phospho-p70 S6 Kinase (Thr389); and Ub-K63, K63-linkage specific polyubiquitin. (**C**) The MV4-11 cells were designated as the control (Cont.) and treated with 0.2 µM of b-AP15 for the indicated times. Subsequently, the cells were treated with (**i**) 5 µg/mL of actinomycin D (Act D) or (**ii**) 50 µg/mL of cycloheximide (CHX) for the indicated time, and a Western blot analysis was performed. (**D**) The cells were cultured in the absence (control) or presence of 0.15 µM of b-AP15 for 4 h. In the presence or absence of b-AP15, no additional substances were added, and the culturing was performed in combination with 10 mM of NAC. The proteins bound to the γ-aminophenyl-m^7^GTP (C10-spacer)-agarose (m^7^-GTP) and whole-cell lysate (TCL) were subjected to cap-binding assays to analyze the eIF4E-eIF4G complex formation. Abbreviations: 4EBP1-nonP: non-phosphorylated 4E-BP1 (Thr46); 4EBP1-P: phosphorylated 4E-BP1; and β-actin: beta-actin.

We speculated that DUB inhibition would elicit various stress responses owing to proteolytic system overload; therefore, we searched for these responses in the FLT3-ITD-positive MV4-11 cell line. In the FLT3-ITD-positive MV4-11 cells, the tb-AP15 treatment caused a concentration-dependent increase in ubiquitinated proteins, AMPK phosphorylation, Mcl-1 attenuation, and PARP cleavage. The intracellular accumulation of the transcription factor Nrf2 was also observed, and both reactions were inhibited by NAC. However, the NAC treatment did not affect the increase in ubiquitinated proteins, AMPK phosphorylation, and the PARP cleavage induced by bortezomib (Figure 2(Ai)). In the AUR treatment group, increased p38 phosphorylation and Nrf2 accumulation were observed (Figure 2(Aii)). In the b-AP15 treatment group, the K48-Ub levels increased over time, with increases in p38-P both before and after the AMPK-P levels increased. Both reactions were prevented by NAC pretreatment (Figure 2(Aiii)). The inhibition of Nrf-2 accumulation by the p38 inhibitor SB203580 occurred in the MV4-11 cells treated with AUR and b-AP15 (Figure S2). In this study, FLT3 expression was reduced following the treatment with USP14/UCHL5 inhibitors and p70S6K signaling downstream of FLT3 was blocked (Figure 2(Bi)). Furthermore, the ubiquitin chains were predominantly composed of K48-Ub, with minimal K63-Ub binding, indicating that the K48-Ub chains bind directly to FLT3 and contribute to its downregulation (Figure 2Bii). Subsequently, we examined the effect of b-AP15 on the FLT3 protein levels following transcriptional inhibition to gain insight into the regulatory mechanisms of FLT3 expression using the transcription inhibitor actinomycin D (Act D) and translation inhibitor cycloheximide. Our findings revealed that the FLT3 levels declined with extended Act D treatment, reaching a low point at 8 h (Figure 2(Ci)). Although there is theoretically residual FLT3 mRNA after transcriptional silencing, adding Act D before b-AP15 resulted in a more rapid decline in FLT3 levels over time than in the cells treated with Act D alone. When Act D and b-AP15 were administered concurrently, the concentration was marginally higher than in the cells treated with b-AP15. In the quantitative polymerase chain reaction experiment, the treatment with b-AP15 had no discernible impact on the FLT3 mRNA levels in the MV4-11 cells. In contrast, b-AP15 did not accelerate the time-dependent decrease in FLT3 levels induced by the cyclohexamide treatment, indicating that b-AP15 does not affect the post-translational step within the specified treatment period (Figure 2(Cii)). These results indicate that b-AP15 affects FLT3 downregulation at the post-transcriptional and translational levels. To understand this regulation of translation levels, we examined the effects on the cap-dependent translation of mRNAs regulated by mTORC1 downstream of FLT3 signaling via 4EBP1 phosphorylation. First, we investigated the impact of b-AP15 on the formation of the eIF4E-eIF4G complex [35,36], crucial for cap-dependent translation, through a pull-down assay utilizing m^7^-GTP beads. The results demonstrated that the b-AP15 treatment reduced phosphorylated 4EBP1 and increased non-phosphorylated 4EBP1 in the MV4-11 cells. These effects were reversed by NAC. In addition, the b-AP15 treatment slightly decreased the eIF4E binding to m^7^-GTP, which led to a notable reduction in eIF4G binding. The pretreatment with NAC markedly inhibited the reduction in the eIF4G binding to m7-GTP-bound eIF4E induced by b-AP15 or AUR (Figure 2D). These findings suggest that the b-AP15 or AUR treatment stabilizes 4EBP1 in the MV4-11 cells, potentially inhibiting the formation of the eIF4E/eIF4G complex required for cap-dependent mRNA translation, including Mcl-1, via a mechanism that is preventable by NAC.

### 2.3. b-AP15 and AUR Decrease the Mitochondrial Membrane Potential, with Apoptosis Inhibited by the Forced Expression of the Mitochondrial Proteins Bcl-xL and Mcl-1

**Figure 3:**
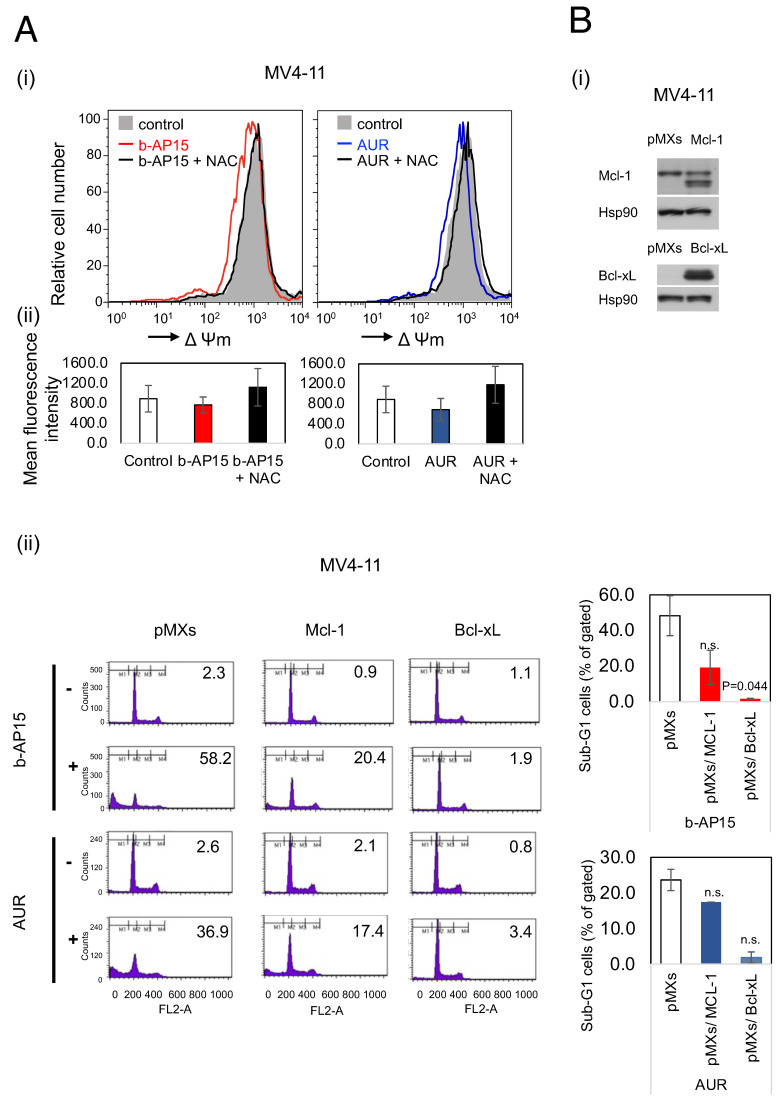
b-AP15 and auranofin (AUR) decrease the mitochondrial membrane potential, with apoptosis inhibited by the forced expression of the mitochondrial proteins Bcl-xL and Mcl-1. (**Ai**) The MV4-11 cells were cultured for 30 min in the absence (control) or presence of 0.15 µM of b-AP15 or 0.3 µM of AUR, with or without 1 mM of NAC. The mitochondrial membrane potential (Δψm) was analyzed by flow cytometry using the DiOC6 fluorescent probe. (**Aii**) Similar experiments were repeated three times. The columns indicate the means and the error bars indicate the standard errors. The statistical analysis was performed using the mean fluorescence intensity values for each group. The Bonferroni multiple comparison test was employed to assess the differences between the other cell groups and the b-AP alone or AUR alone treatment groups. (**Bi**) The MV4-11 cells with forced expression of Mcl-1 or Bcl-xL and the vector control cells (pMXs) were cultured in Iscove’s Modified Dulbecco Medium (IMDMc) and analyzed via immunoblotting with the indicated antibodies. (**Bii**) The MV4-11 cells transformed with Mcl-1, Bcl-xL, or the vector control (pMXs) were treated with b-AP15 or AUR for 20 h. The cellular DNA content was analyzed by flow cytometry, and the percentage of apoptotic cells with sub-G1 DNA content was indicated. The differences in other cell groups relative to the vector control group were tested using Dunnett’s multiple comparison method. n.s. indicates not significant.

Treating MV4-11 cells with b-AP15 or AUR reduced the mitochondrial membrane potential, an effect completely negated by NAC treatment, indicating that intracellular ROS fluctuations mediate USP14-induced apoptosis (Figure 3A). A key downstream target of the FLT3-activated mTOR/4EBP1/eIF4E pathway is Mcl-1, an intramitochondrial anti-apoptotic protein in the Bcl-2 family. This highly unstable protein requires active cap-dependent translation to maintain its expression level. The observed reduction in the mitochondrial membrane potential by b-AP15 and AUR (Figure 3A) likely occurs alongside the activation of the endogenous mitochondrial pathway (Figure 1B). Therefore, we investigated the impacts of the forced expression of Bcl-xL and Mcl-1 on b-AP15- and AUR-induced apoptosis.

### 2.4. USP14 Knockdown Increases Sensitivity to USP14 Inhibitors and Induces Apoptosis in Leukemia Cells from Patients with FLT3-ITD-Positive AML

**Figure 4:**
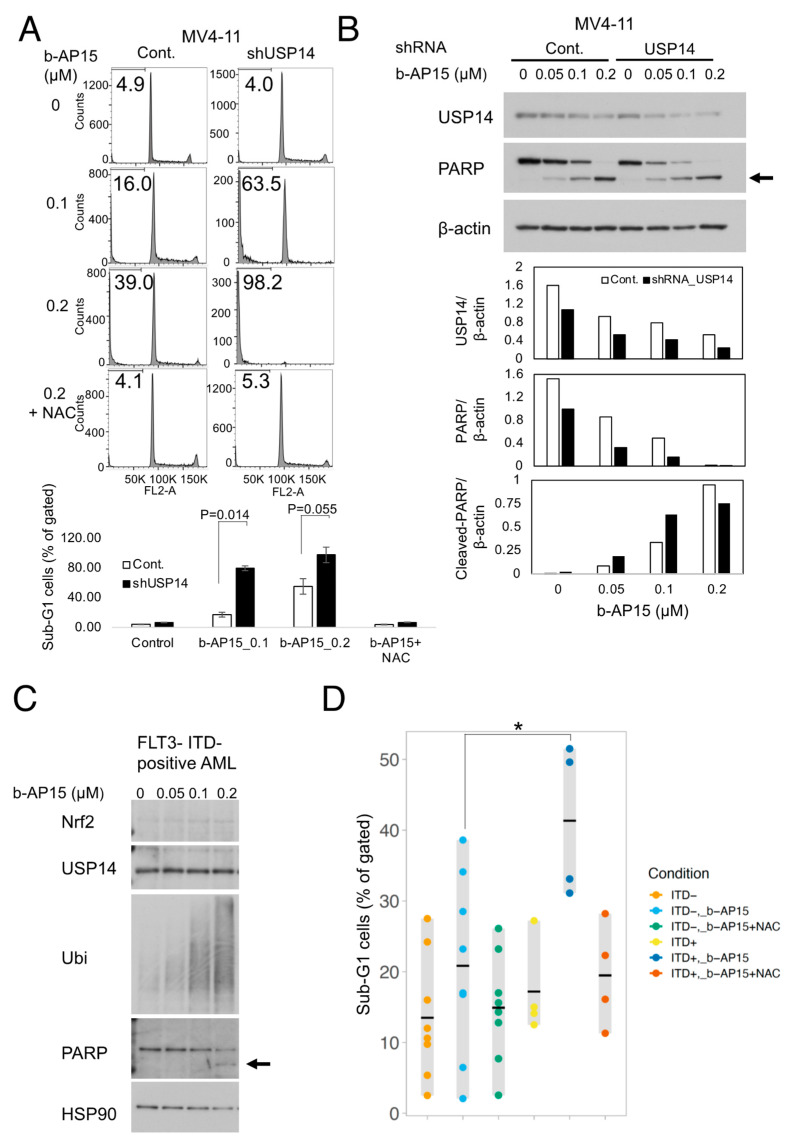
USP14 knockdown increases sensitivity to USP14 inhibitors and induces apoptosis in leukemia cells from patients with FLT3-ITD-positive AML. Abbreviations: FLT3, FMS-like tyrosine kinase 3; AML, acute myeloid leukemia; and ITD, internal tandem duplication. (**A**) The MV4-11 cells with knockdown of USP14 (shUSP14) or vector control cells (the Cont.) were cultured for 24 h in the absence (control) or presence of 0.1 µM or 0.2 µM of b-AP15. b-AP15 was incubated with or without 10 mM of NAC. The cellular DNA content was analyzed by flow cytometry, and the percentage of apoptotic cells with sub-G1 DNA content was documented. Similar experiments were conducted four times, with the columns representing the means and the error bars representing the standard errors. Welch’s *t*-test (two-tailed) was employed to assess the differences between the MV4-11 cells with the knockdown of USP14 (shUSP14) and the vector control cells (the Cont.). (**B**) The MV4-11 cells with USP14 knockdown (USP14) or vector control cells (the Cont.) were treated with b-AP15 for 5 h and analyzed via immunoblotting with the indicated antibodies. The arrow indicates the level of cleaved PARP. The concentration of the individual bands was quantified using densitometry. (**C**) Primary AML cells derived from cases with FLT3-ITD were designated as the controls and left untreated or treated with 0.05, 0.1, or 0.2 µM of b-AP15 for 5 h, after which they were lysed. The indicated antibodies were used for the immunoblot analysis. The arrow indicates the level of cleaved PARP. (**D**) Primary AML cells derived from patients with FLT3-ITD-positive (*n* = 4) or -negative (*n* = 8) AML were cultured for 6 h in the absence or presence of 0.2 µM of b-AP15. The cells were incubated for 6 h in the absence (control) or the presence of b-AP15. The cellular DNA levels were analyzed by flow cytometry. The percentage of apoptotic cells with sub-G1 DNA content for each case is indicated by each point; the mean value for each group is indicated by the bars; and the range of each point is indicated by the vertical bars. The asterisks indicate statistically significant differences by Welch’s *t*-test (two-tailed) (*p* < 0.05).

The impact of USP14 knockdown on FLT3-ITD cells was investigated, and the results showed that USP14 knockdown rendered these cells more susceptible to b-AP15-induced apoptosis (Figure 4A). The MV4-11 cells with USP14 knockdown exhibited heightened sensitivity to b-AP15-induced PARP cleavage (Figure 4B). Studies of leukemic cells from patients with FLT3-ITD-positive AML revealed augmented Nrf2 accumulation, PARP cleavage, and elevated ubiquitination, as observed in the cell lines (Figure 4C). Moreover, the induction of apoptosis was markedly more pronounced in these cells than in ITD-negative cells (Figure 4D).

## 3. Discussion

This study demonstrated that the USP14/UCHL5 inhibitors b-AP15 and AUR effectively inhibited the K48-mediated deubiquitination of ubiquitin bound to FLT3 in leukemia cells with FLT3-ITD mutations. This inhibition resulted in the downregulation of FLT3, inhibiting its downstream signaling and the formation of a 4EBP1-mediated translation initiation complex. These findings suggest that these combined mechanisms induced oxidative stress and apoptosis in the leukemia cells.

USP14 acts on various cells and is involved in deubiquitination processes [13]. Apoptosis induction in leukemia cells through USP14 inhibition has been previously documented [37]; however, this is the first study to identify a specific mechanism by which USP14/UCHL5 inhibition induces apoptosis in FLT3-ITD-positive AML cells or in primary FLT3-ITD-positive AML cultures. In the UPS, the K11- and K48-Ub chains target intracellular misfolded proteins for proteasomal processing [38], whereas the K63-Ub chains target protein aggregates and dysfunctional intracellular organelles for degradation by the autophagy–lysosome pathway [39].

**Figure 5:**
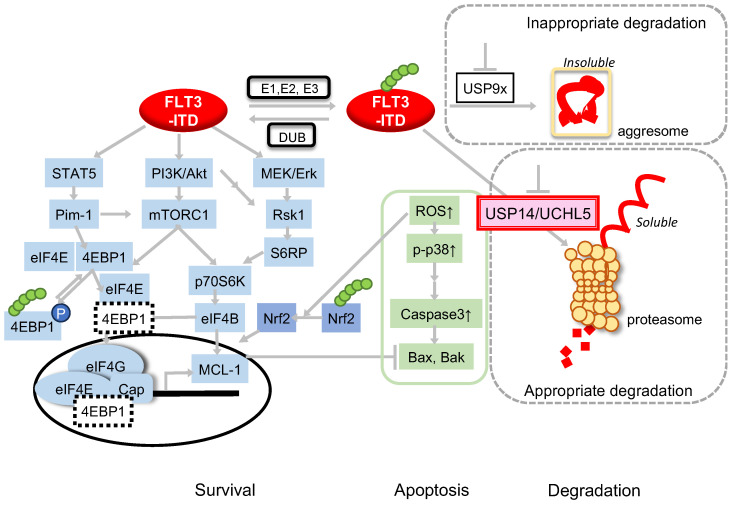
Schematic model of the intracellular signaling mechanism regulating deubiquitinating enzyme inhibitor-induced apoptosis in FLT3-ITD, involving the STAT5/Pim axis and the mTORC1/eIF4E/Mcl-1 and mitochondrial-intrinsic pathways. The arrows show the direction of the signal. Circles indicate the nucleus. The dotted box indicate proteins that are present both inside and outside the nucleus. The green circle indicates Ubiquitin.

Our previous study demonstrated that USP9X inhibition in FLT3-ITD-positive AML induced the K63-Ub processing of receptor-type tyrosine kinases to form aggresomes [34], suggesting a rapid removal mechanism for the misfolded proteins in leukemia cells. However, in this study, USP14/UCHL5 inhibition by b-AP15 did not result in the formation of insoluble proteins, indicating a different mechanism of action. We hypothesize that USP14/UCHL5 plays a significant role in the context-specific proteolytic process. Although b-AP15 is a specific inhibitor of USP14/UCHL5, AUR, a nonspecific inhibitor of DUBs, may exert other effects. The effects observed with b-AP15 were consistently found around 0.2 μM, and USP14 knockdown increased the sensitivity of MV4-11 cells to apoptosis by b-AP15, highlighting the importance of the inhibition of USP14 alone or USP14/UCHL5 for the FLT3-ITD downregulation and apoptosis induction. The impact of FLT3-ITD on ubiquitination was observed with both pharmacological agents, proving that USP14/UCHL5 inhibition plays a role in this outcome. However, a detailed examination of the relationship between the effects of AUR and its concentration indicated that the increase in the Ub-activated protein (Figure 2B) is relatively minor, even at high concentrations of the AUR treatment. This suggests that other mechanisms beyond DUB inhibition may contribute to the apoptosis-inducing and p38-phosphorylation effects of AUR observed at lower concentrations. For example, the effect of TrxR should be considered in conjunction with other mechanisms, such as the inhibition of the JAK/STAT and PI3K/Akt/mTOR pathways, which may also contribute to the observed inhibition of proliferation. Our previous study showed that c-Cbl and Cbl-b, E3 ubiquitin ligases of the RING family, induced K48-mediated polyubiquitination of autophosphorylated ITD, thereby promoting its degradation through the lysosomal and proteasomal systems [33]. Conversely, a recent report identified NEDD4 as the E3 ligase involved in K63-mediated ITD polyubiquitination [40]. These findings indicate that USP14/UCHL5 and USP9X may regulate deubiquitination via different E3 ligases. Therefore, identifying the functional distinctions between the respective DUB-mediated FLT3 ubiquitination in the future is essential. The results in this study indicate that b-AP15 inhibited the formation of the eIF4E/eIF4G complex necessary for cap-dependent mRNA translation through a mechanism that can be prevented by NAC. It is necessary to discuss the relationship between the deubiquitination of the FLT3-bound ubiquitin and the mTOR signaling pathway. Two distinct mechanisms may be involved. First, the FLT3 downregulation results in the attenuation of homeostatically activated signaling, which, in turn, affects the downstream mTOR-4EBP1 axis. The data presented in Figure 2(Bi) provide evidence to support this assertion. In this study, FLT3 expression was reduced following the treatment with USP14/UCHL5 inhibitors and blocked p70S6K signaling downstream of FLT3. Second, 4EBP1 is subject to regulation by the ubiquitin system. Although the hypothesis is untested, the apparent inhibitory effect of USP14/UCHL5 inhibitors on the transcription initiation complex formation in this study suggests that USP14/UCHL5 may be involved in this ubiquitination. Further research is needed to understand the pathophysiology and regulation of AML and other malignancies. This study demonstrated that USP14/UCHL5 inhibition by b-AP15 or AUR attenuates Mcl-1 expression, consistent with previous reports [41,42,43], which implicated the reduced Mcl-1 expression in USP14/UCHL5 inhibition, resulting in apoptosis. Furthermore, the apoptosis induced by the USP14/UCHL5 inhibitors b-AP15 and AUR was prevented by forced Mcl-1 expression. FLT3 downregulation delays protein digestion and inhibits the formation of the translation initiation complex. Our findings indicate that USP14 inhibitors induced apoptosis in ITD-positive cells by simultaneously blocking ITD signaling and triggering oxidative stress. A previous report demonstrated that FLT3-ITD elevates ROS production, and FLT3-ITD-positive cells are susceptible to USP14/UCHL5 inhibition [44]. The precise mechanism remains unclear; however, DUBs may play a role in mitochondrial quality control, and its disruption may increase ROS, resulting in oxidative stress [45,46]. The accumulation of Nrf2 observed in this study suggests an association with increased ROS. However, the levels of the Nrf2 protein also depend on Ub/proteasome degradation. Therefore, further analysis is warranted to determine the relationship between the accumulation of Nrf2 and the DUB inhibitory activity of both drugs. As previously described, ITD-positive AML cells depend on efficient protein degradation mechanisms, including the USP14/UCHL5-mediated degradation of large amounts of intracellular proteins in the UPS and antioxidant regulation (Figure 5). Therefore, targeting this mechanism could effectively disrupt FLT3-positive AML homeostasis, which is difficult to control.

This study had some limitations. First, the retrospective nature of the clinical sample data may have introduced selection bias and unmeasured confounding factors, which could have affected the results. Second, the study population was heterogeneous, and the number of patients evaluated was small, which may have introduced bias. Third, this study employed a single cell line, MV4-11, which is the most prevalent cell line with FLT3-ITD mutations. However, the available cell lines were limited. Furthermore, as is the case with studies that employ clinical specimens and cell lines, the presence of other significant genetic mutations, such as *NPM-1* and *p53*, was not evaluated. Further studies with larger sample sizes and more diverse cell lines are necessary to validate these findings and address potential confounding factors.

## 4. Materials and Methods

### 4.1. Cell Culture and Reagents

The MV4–11 cells (CRL-9591) were purchased from the American Type Culture Collection (Manassas, VA, USA) and cultured in Iscove’s Modified Dulbecco Medium (IMDM, WAKO098-06465, FUJIFILM Wako Chemicals, Osaka, Japan) containing 10% fetal calf serum (FCS, Nichirei, 175012). The human leukemic cell lines HEL, K562, and U937 were obtained from the Riken Cell Bank (Ibaraki, Japan) and cultured in Rosewell’s Park Memorial Institute 1640 medium containing 10% FCS. The amphotropic virus packaging cell line PLAT-A [47] and the human embryonic kidney cell line 293T [48] were provided by Toshio Kitamura and Shoji Yamaoka, respectively, and cultured in Dulbecco’s Modified Eagle’s Medium (SHIMADZU, 05919) containing 10% FCS. The deubiquitinase inhibitors b-AP15 (#11324, cas #1009817-63-3) and AUR (SC-202476) were procured from Cayman Chemical (Ann Arbor, MI, USA) and Santa Cruz Biotechnology (Dallas, TX, USA), respectively. The bortezomib was sourced from LC laboratories (Woburn, MA, USA). The m^7^-GTP-Agarose (AC-155S) was obtained from Jena Bioscience (Jena, Germany). Act D (cat. no. GR-300) was obtained from BIOMOL (Humburg, Germany), and 3,3′-dihexyloxacarbocyanine iodide (DiOC6) was purchased from Invitrogen (Carlsbad, CA, USA). The following reagents were purchased from Sigma-Aldrich (St Louis, MO, USA): propidium iodide (PI), cycloheximide, doxycycline, tBHQ (11291), and NAC. The antibodies against FLT3 (SC-479), HSP90 (sc-13119), and ubiquitin (Ub) (sc-8017) were purchased from Santa Cruz Biotechnology. An anti-Bax monoclonal antibody (clone YTH-6A7, cat#2281-MC-100) was sourced from Trevigen (Gaithersburg, MD, USA). The antibodies against PARP (BML SA-250), the K63- linkage-specific Ub (K63-Ub), and β-actin (A1978), were purchased from Enzo Life Sciences (Farmingdale, NY, USA) and Sigma-Aldrich, respectively. The antibodies against Nrf2 (#12721), phospho-AMPK (Thr172) (#2535), phospho-70S6K-TP (Thr389) (#9234), phospho-FLT3 (Tyr591) (#3461), cleaved Caspase-3 (#9661), Caspase-9 (#9508), cleaved Caspase-9 (#9509), phospho-4EBP1 (Thr37/46) (#2855), phospho-4EBP1-S65 (#9451), non-phospho-T46–4EBP1 (#4923), phospho-p38 MAP Kinase (Thr180/Tyr182) (#9211), Mcl-1 (#5453), eIF4E (#2067), and eIF4G (#2469I), were purchased from Cell Signaling Technology (Beverly, MA, USA). The K48-linkage-specific Ub (K48-Ub) rabbit mAb was sourced from ABclone (A3606), and the anti-USP14 antibody (67746-1-Ig) was procured from Proteintech (Rosemont, IL, USA). The various inhibitors were dissolved in dimethyl sulfoxide and added to the culture medium at a final concentration of <0.01%.

### 4.2. Expression of the Plasmids, Transfection, and Infection

The expression of the plasmids, transfection, viral infection, and creation of retroviral expression plasmids for Bcl-xL and Mcl-1, namely, pMXs-puro-Bcl-xL and pMXs-puro-Mcl-1, were performed as previously described [6,49]. The MV4-11 cells overexpressing Mcl-1 were obtained by infection with pMXs-puro or pMXs-puro-Mcl-1, which served as the controls, and subsequent selection with puromycin. For the gene expression repression, pLKO.1-puro-GFP-siRNA, a gift from Bob Weinberg (Addgene plasmid #12273) [49], and shUSP14 (Merck, predesigned shRNA, TRCN0000007427) were used as previously described [50,51]. The lentiviral packaging plasmid, psPAX2, and envelope expressing plasmid, pMD2.G, were gifts from Didier Trono (Addgene plasmids #12260 and #12259).

### 4.3. Analysis Using Flow Cytometry

The procedures for the flow cytometric analysis of the cell cycle and apoptosis, mitochondrial membrane potential, Bax conformational changes, and caspase-3 cleavage were performed as previously described [27]. For the flow cytometric analysis of the cell cycle and apoptosis, the cells were treated with Krishan’s reagent (0.05 mg/mL of PI, 0.1% sodium citrate, 0.02 mg/mL of ribonuclease A, and 0.3% NP-40) for 30 min on ice and analyzed by flow cytometry. The flow cytometric analyses of Bax conformational changes and caspase-3 cleavage were performed using specific antibodies against activated Bax and cleaved caspase-3 [49]. For the flow cytometric analysis of Δψm, the cells were stained with DiOC6 and PI and analyzed as previously described [49]. The intracellular ROS levels were measured as previously described [52]. All the flow cytometric analyses were conducted using the BD FACSCalibur (BD Biosciences) or MAQSQuant Analyzer 10 (Milteny Biotec, Bergisch Gladbach, Germany) and analyzed using FlowJo 10.10.0 (BD Biosciences, Franklin Lakes, NJ, USA).

### 4.4. Immunoblotting, Cap-Binding Assays, and Immunoprecipitation

The cells were lysed and subjected to an immunoblot analysis as described above [6,33]. The cells were lysed in a solution of 1% Triton X-100, 20 mM of Tris-HCl (at a pH of 7.5), 150 mM of NaCl, 1 mM of EDTA, 1 mM of sodium orthovanadate, 1 mM of phenylmethylsulfonyl fluoride, and 10 μg/mL each of aprotinin and leupeptin. An aliquot of the cell lysate was mixed with an equal volume of the Laemmli sample buffer and heated at 100 °C for 5 min. Subsequently, the samples were subjected to immunoprecipitation and immunoblotting. For the cap-binding experiments, the cell lysates were incubated overnight at 4 °C with γ-aminophenyl-m^7^GTP (C10-spacer)-Agarose (AC-155S, Jena Bioscience, Jena, Germany). The beads were extensively washed with the lysis buffer and heated at 100 °C for 5 min in 1× Laemmli’s buffer. The eluted proteins were then subjected to the immunoblot analysis.

### 4.5. Analyses of Primary AML Cells

The cryopreserved samples from 12 patients diagnosed with AML at Tokyo Medical and Dental University Hospital between January 2017 and July 2024, analyzed for FLT3 gene mutations, were used. Specifically, the mononuclear cells were separated from the bone marrow or peripheral blood samples of four FLT3-ITD-positive and eight FLT3-ITD-negative patients with AML by centrifugation using Lymphoprep (PROGEN Biotechnik GmbH, Heidelberg, Germany). The cryopreserved cells were thawed and cultured in IMDM containing 10% FCS for 1 day before analysis. The FLT3-ITD mutations were detected by the reverse transcription polymerase chain reaction as previously described [6] or by the same method in a clinical laboratory. The mononuclear cells were isolated and analyzed for the cell cycle phase by the immunoblot analysis and flow cytometry. This study was approved by the Ethics Committee of Tokyo Medical and Dental University (approval number: M2017-002) in accordance with the Declaration of Helsinki, and written informed consent was obtained from all the patients.

### 4.6. Statistical Analysis

The values are presented as the mean ± standard error, representing two or three independent experiments, each conducted twice. Unless stated otherwise, the Student’s *t*-test was employed to assess the statistical significance of the mean differences between the two groups. A one-way analysis of variance (ANOVA) was utilized to calculate the mean differences among three or more experimental groups, followed by the Dunnett’s test to compare with the control group. The statistical significance was set at 0.05 (*), 0.01 (**), 0.001 (***), and not significant (NS) (*p* > 0.05). All the statistical analyses were performed using Mac Toukeikaiseki Ver. 3.0 (ESUMI Co., Ltd., Tokyo, Japan).

## 5. Conclusions

This study is the first to demonstrate that the USP14/UCHL5 inhibitors b-AP15 and AUR effectively inhibit the K48-mediated deubiquitination of ubiquitin bound to FLT3, resulting in FLT3 downregulation and inhibition of its downstream signaling in FLT3-ITD-positive AML cells. Moreover, the forced expression of the anti-apoptotic proteins Bcl-xL and Mcl-1 prevented the apoptosis induced by b-AP15 and AUR, thereby underscoring the role of these pathways in cell survival. Our study makes a significant contribution to the existing literature on FLT3-ITD in AML, offering novel insights into the molecular mechanisms that regulate the processes contributing to therapeutic resistance in this disease. Furthermore, it highlights the potential of USP14/UCHL5 as a target for therapeutic intervention.

## Data Availability

The original contributions presented in this study are included in this article; further inquiries can be directed to the corresponding author.

## Supplementary Materials

The following supporting information can be downloaded at https://www.mdpi.com/article/10.3390/ijms251910372/s1.
