# Supplementary material for: Targeting USP14/UCHL5: A Breakthrough Approach to Overcoming Treatment-Resistant FLT3-ITD-Positive AML
Source: Int J Mol Sci. 2024 Sep 26;25(19):10372. doi: 10.3390/ijms251910372 (PMC11476563; doi:10.3390/ijms251910372)
Supplement: Supplementary file 1 [file ijms-25-10372-s001.zip › Supplemental figures 2/Supplementary_Materials_for_resubmission4.pdf]

Supplementary Materials

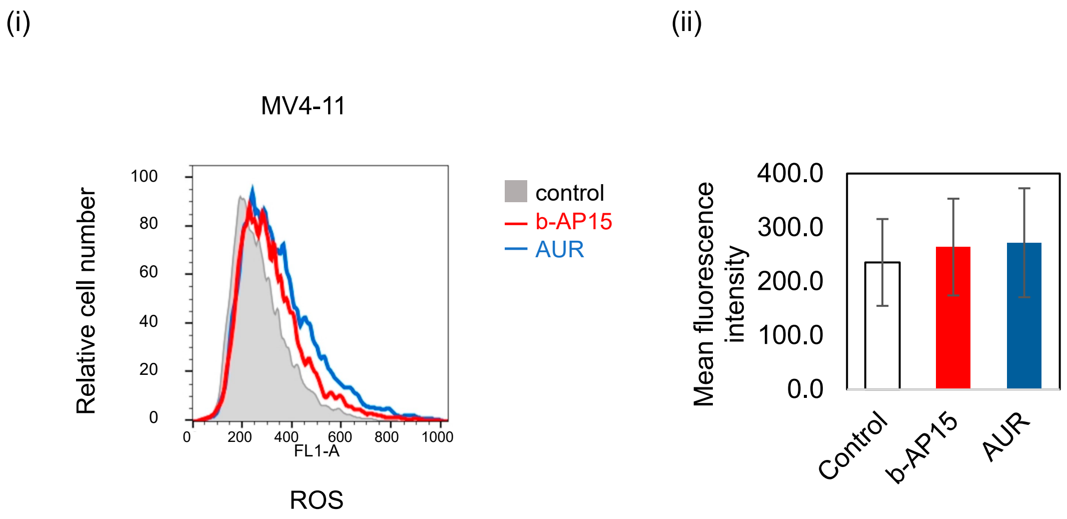

Figure S1. Measurement of reactive oxygen species (ROS) in MV4-11 cells after treatment with b-AP15 or auranofin. (i) MV4-11 cells were either untreated (control) or treated with 0.5 μM auranofin (AUR) and 0.2 μM b-AP15 for 30 min. Cellular ROS were analyzed by flow cytometry using the redox-sensitive fluorescent probe, dichlorofluorescein diacetate. The figure depicts a typical histogram. (ii) Experiments were repeated three times. Columns indicate means and error bars indicate standard errors. Statistical analysis was performed by Dunnett's multiple comparison method using mean fluorescence intensity values for each group.

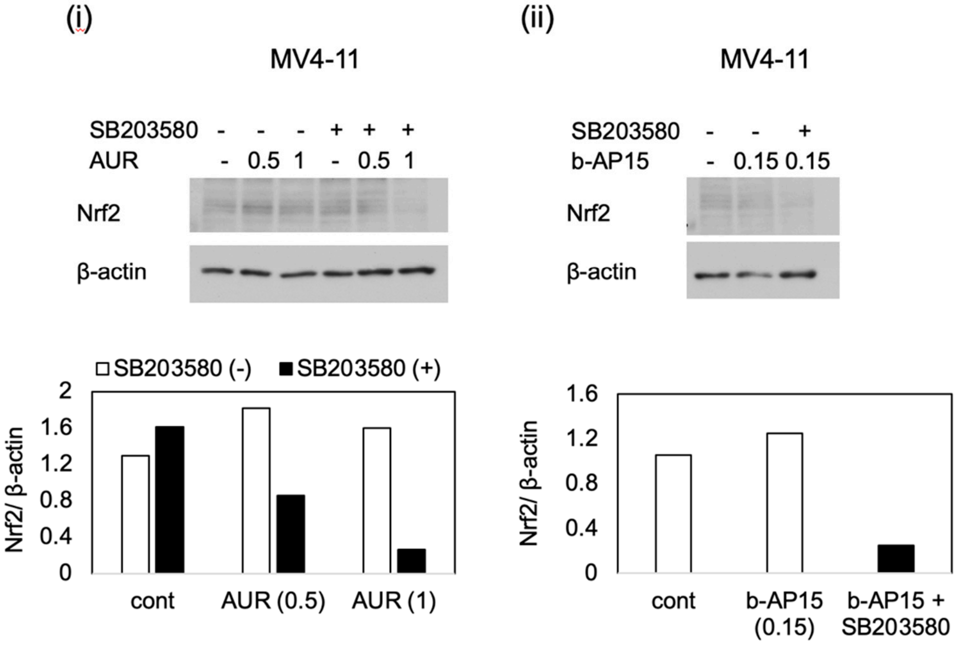

Figure S2. Inhibition of Nrf-2 accumulation by the p38 inhibitor SB203058 in MV4-11 cells treated with (i) Auranofin (AUR) and (ii) b-AP15. Cells were treated with or without 50 μM SB203058 for 5 h, in the absence (control) or presence of 0.15 μM b-AP15, 0.5 μM AUR, or 1 μM AUR. Subsequently, the cells were lysed and subjected to immunoblot analysis using antibodies directed against the indicated proteins. β-actin served as a loading control. Abbreviations: Nrf-2, NF-E2-related factor 2; β-actin: beta-actin.
