# Supplementary figures and images for: Targeting USP14/UCHL5: A Breakthrough Approach to Overcoming Treatment-Resistant FLT3-ITD-Positive AML
Source: Int J Mol Sci. 2024 Sep 26;25(19):10372. doi: 10.3390/ijms251910372 (PMC11476563; doi:10.3390/ijms251910372)

Figure 1B

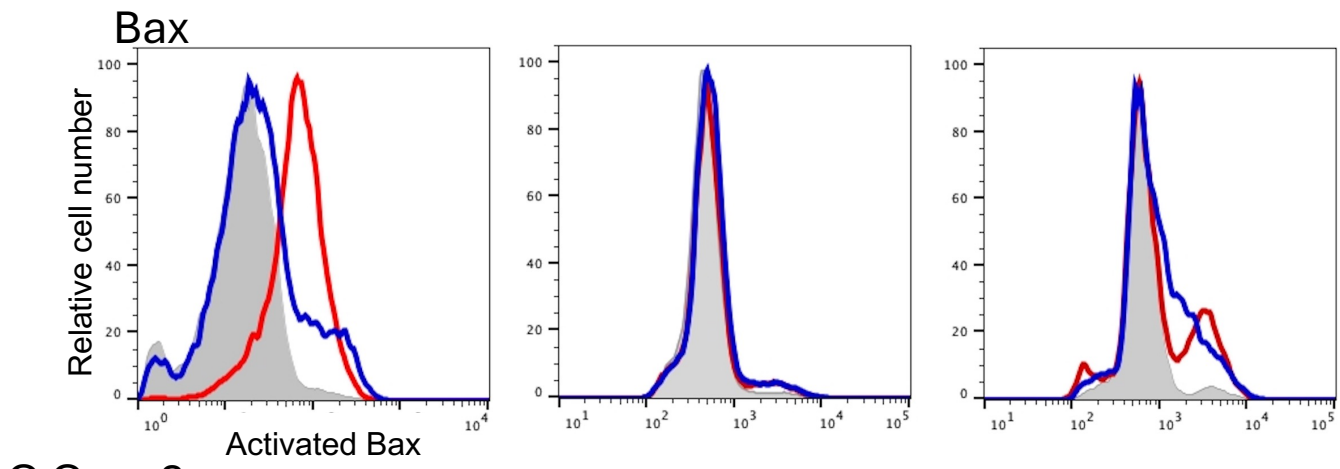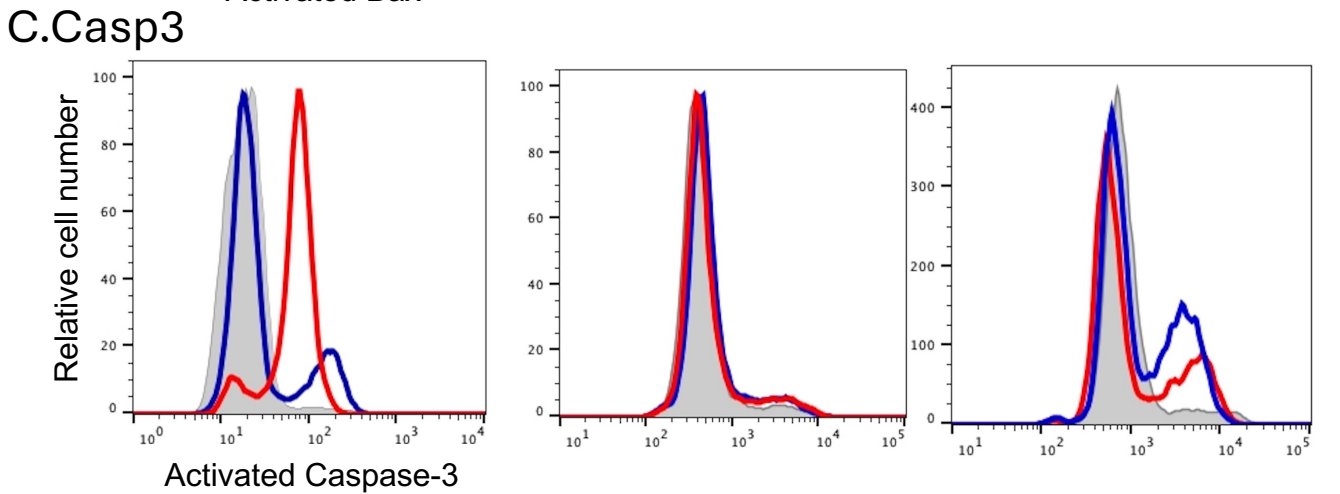

Figure 3A

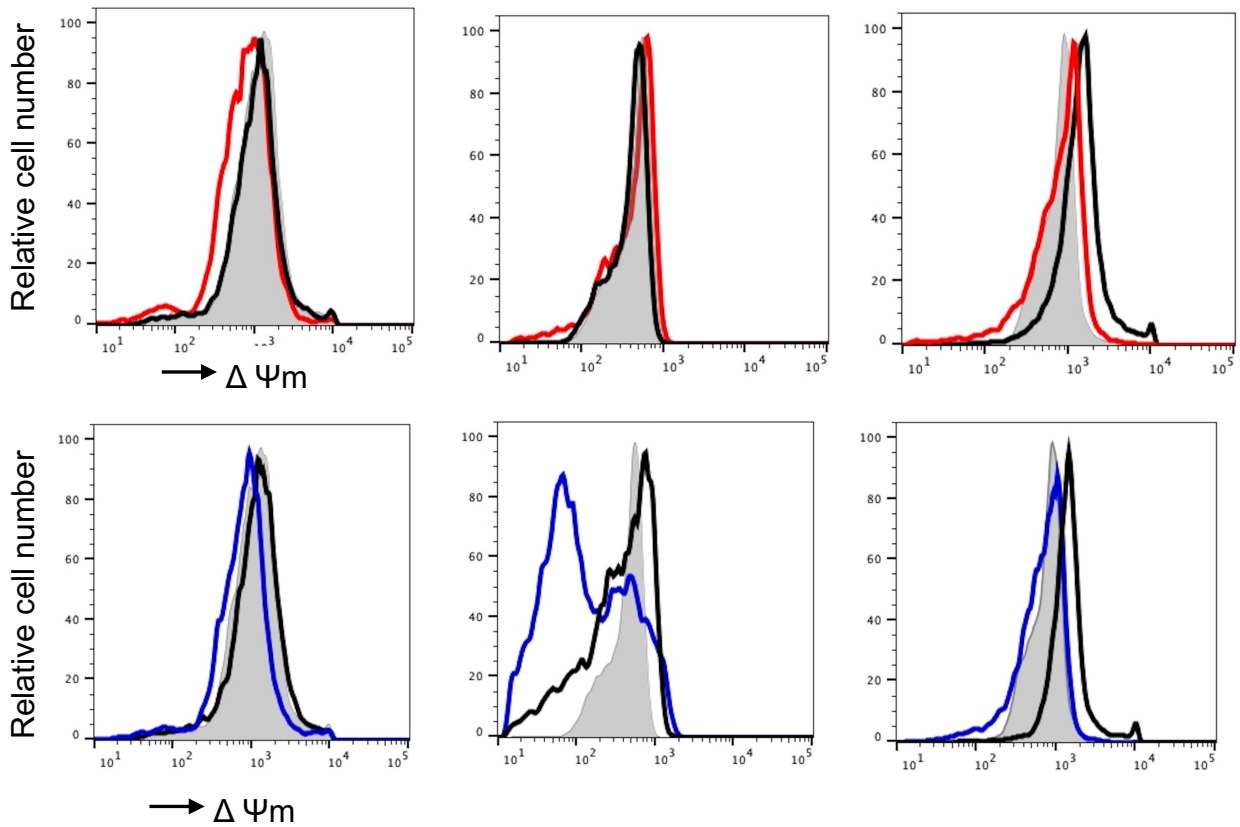

Figure S1

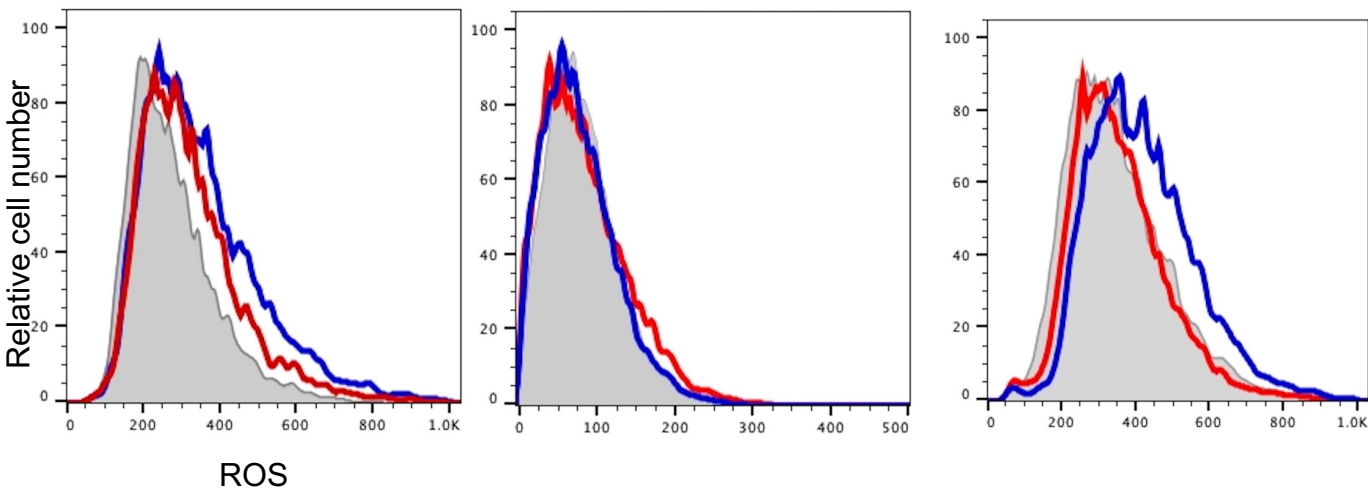

Supplement: Supplementary file 1 [file ijms-25-10372-s001.zip › Supplemental figures 2/Data set of histogram.pdf]

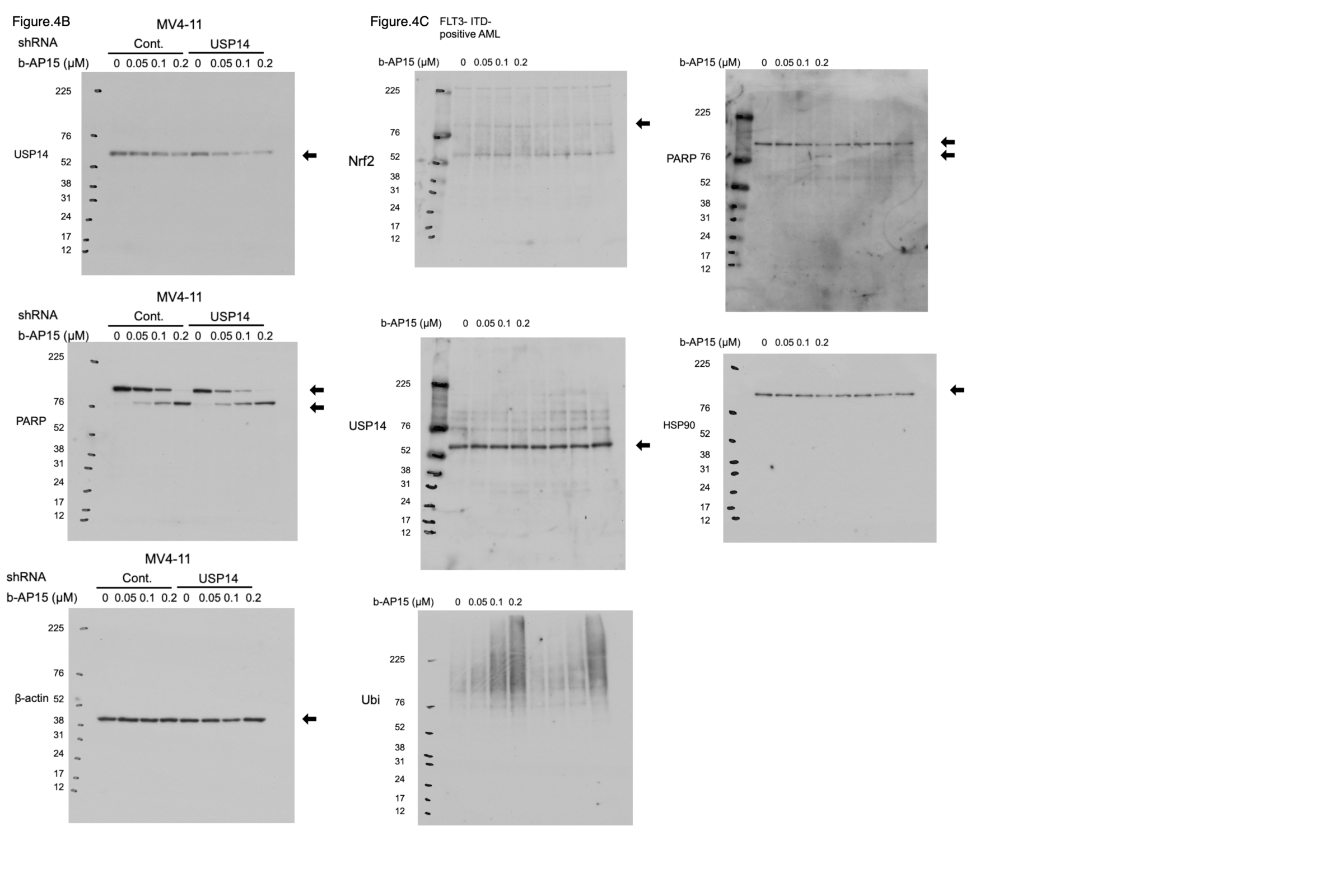

Supplement: Supplementary file 1 [file ijms-25-10372-s001.zip › Supplemental figures 2/original western blot data set1_ds/WholeWB.Data.4B4C-ds.tif]

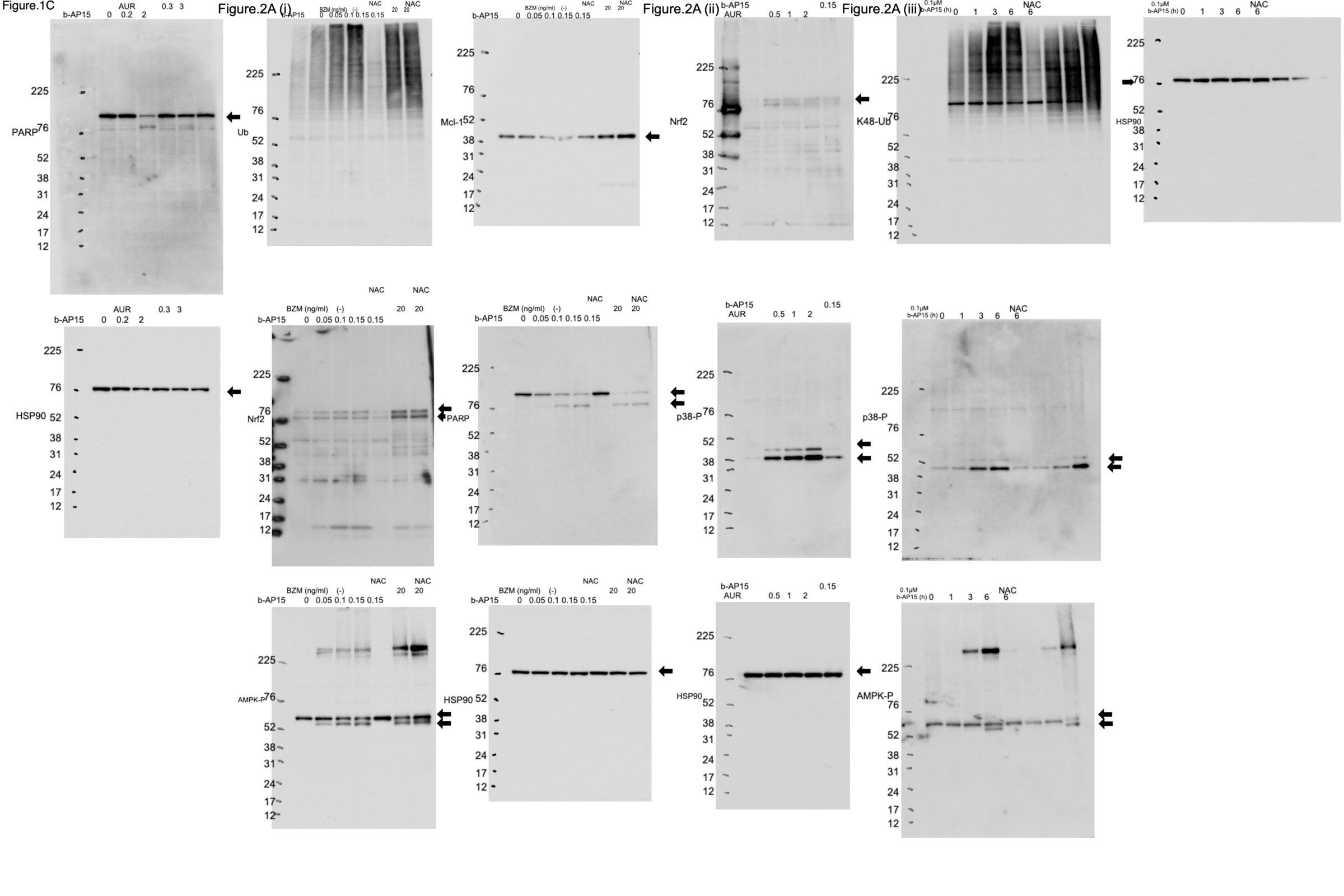

Supplement: Supplementary file 1 [file ijms-25-10372-s001.zip › Supplemental figures 2/original western blot data set1_ds/WholeWB.Data.Fig1C-ds.tif]

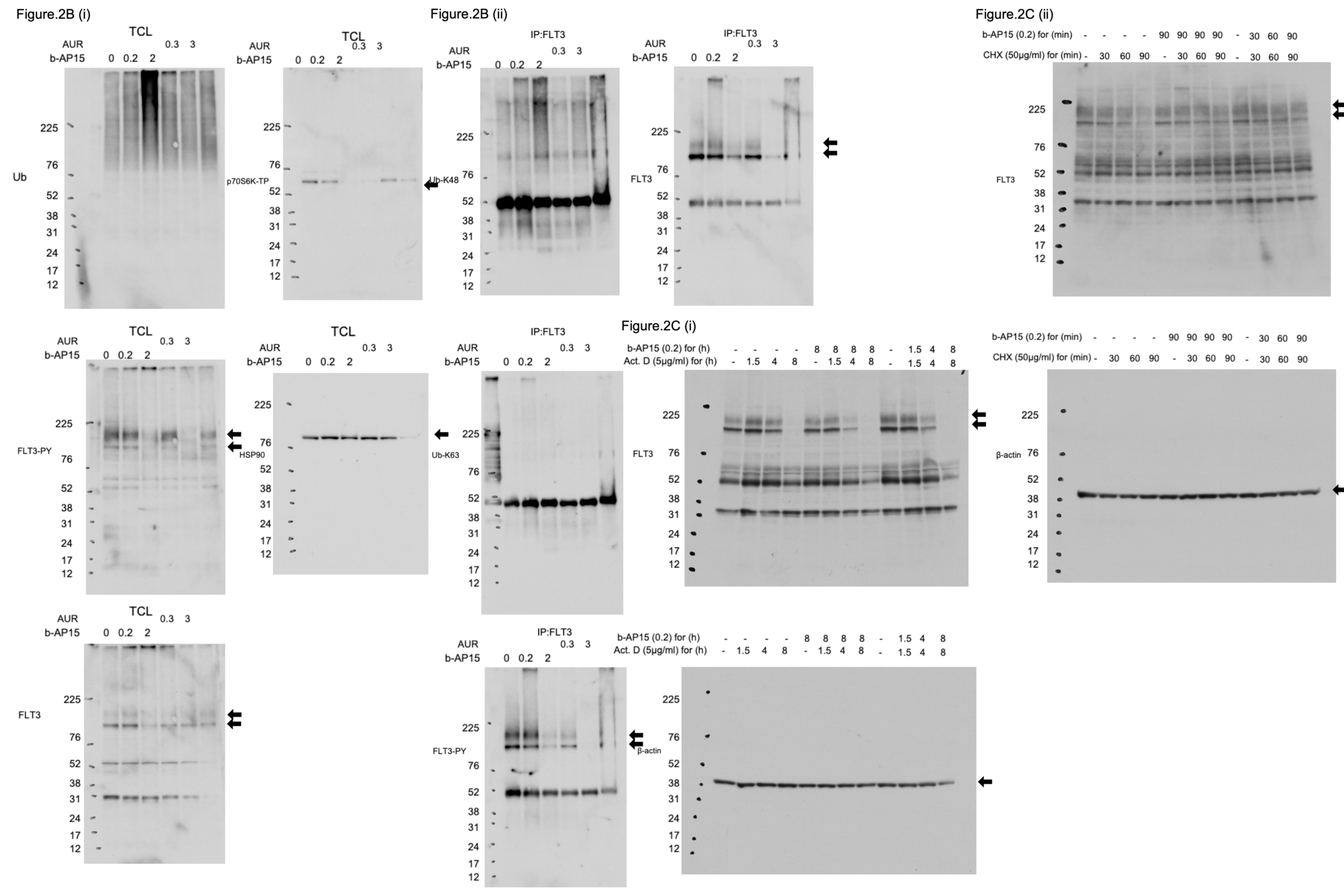

Supplement: Supplementary file 1 [file ijms-25-10372-s001.zip › Supplemental figures 2/original western blot data set1_ds/WholeWB.Data.Fig2B-ds.tif]

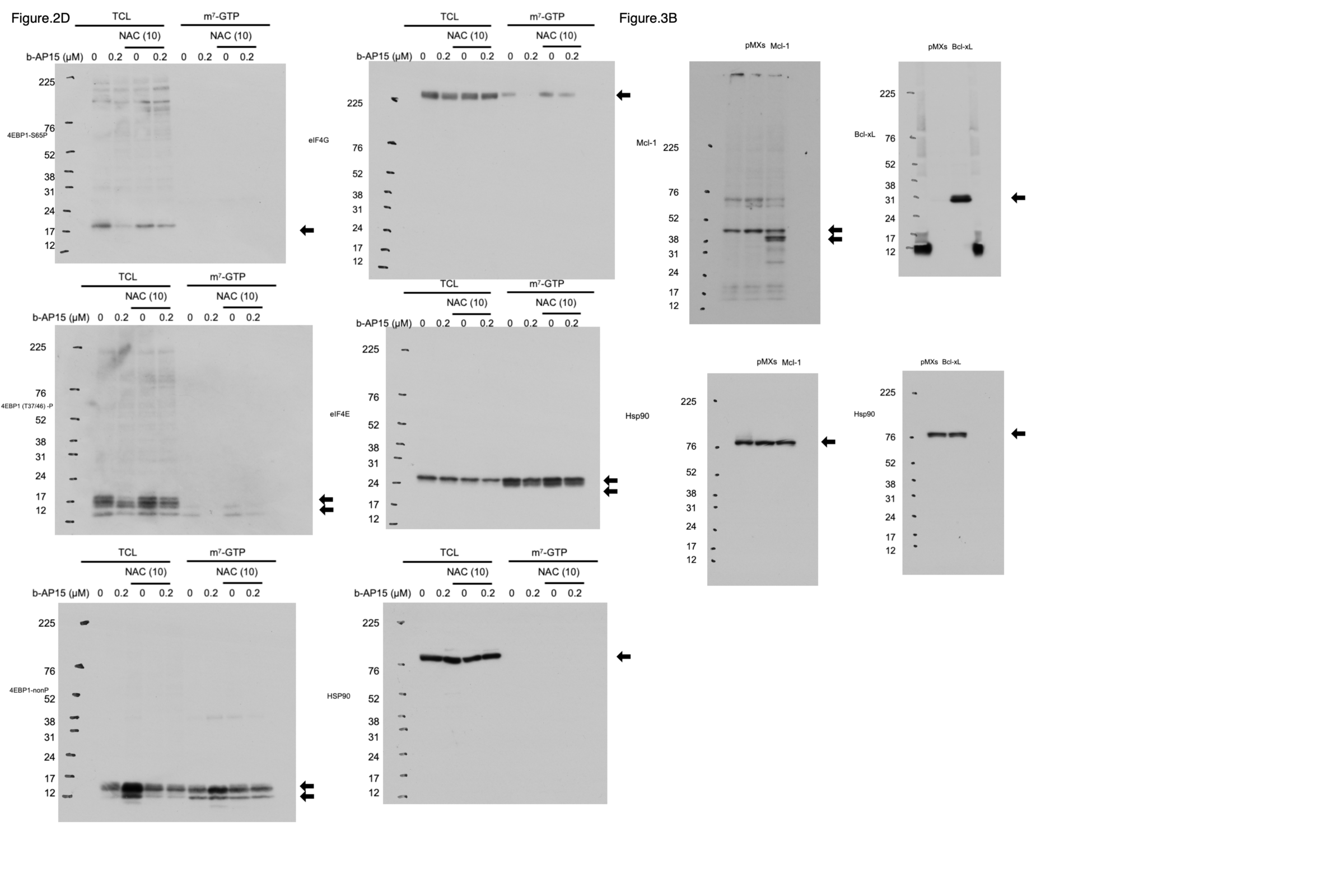

Supplement: Supplementary file 1 [file ijms-25-10372-s001.zip › Supplemental figures 2/original western blot data set1_ds/WholeWB.Data.Fig2D-ds.tif]

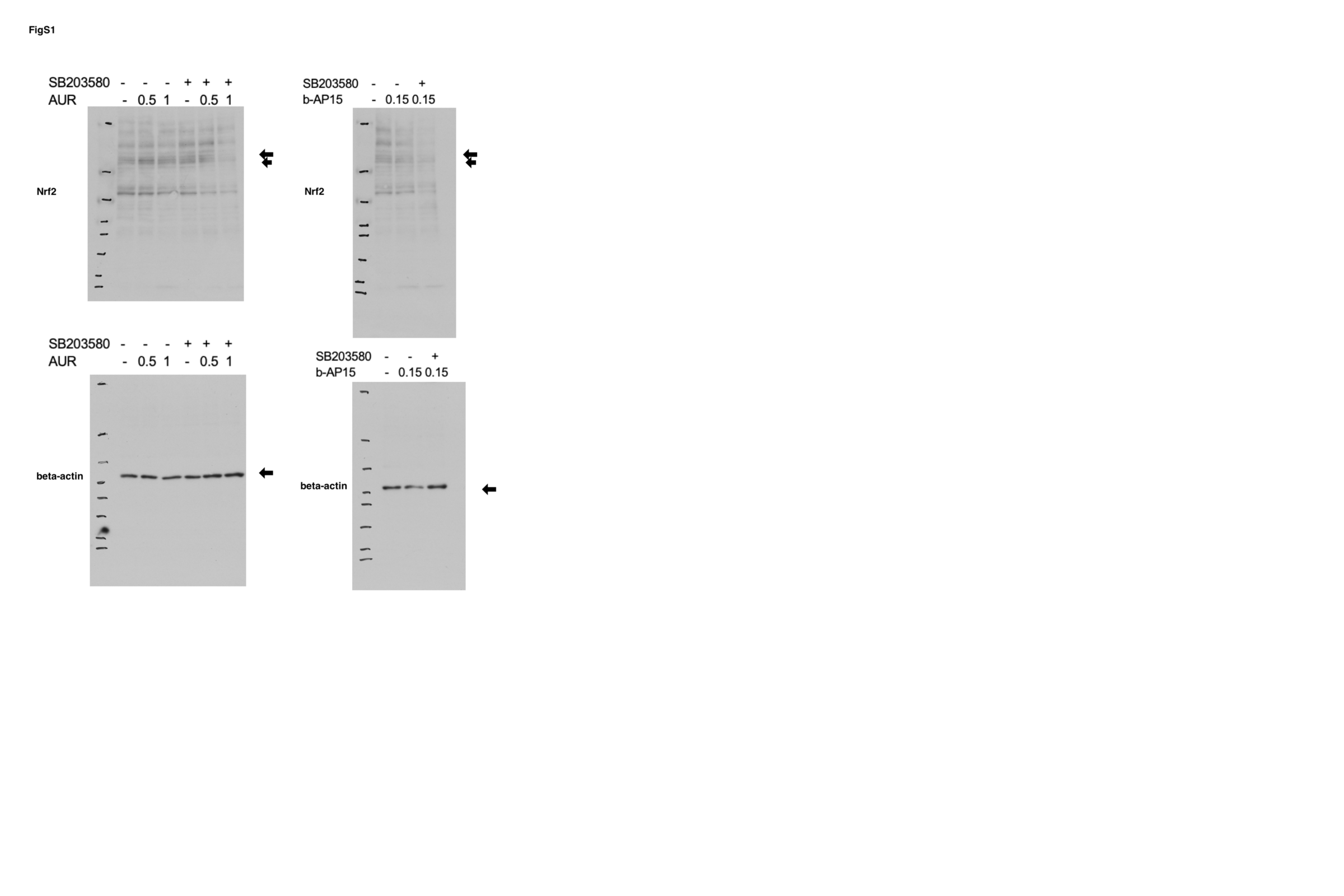

Supplement: Supplementary file 1 [file ijms-25-10372-s001.zip › Supplemental figures 2/original western blot data set1_ds/WholeWB.Data.FigS1-ds.tif]

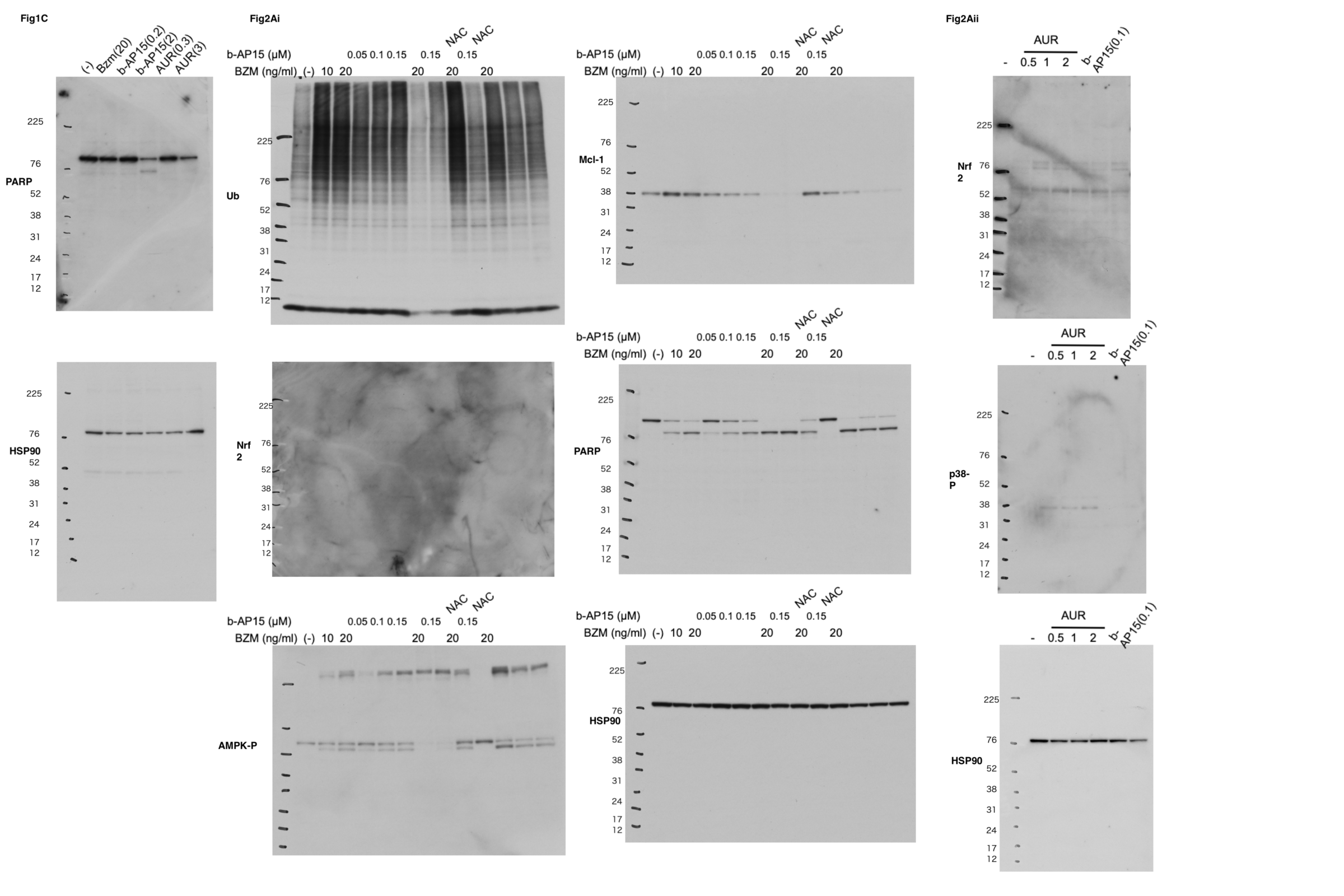

Supplement: Supplementary file 1 [file ijms-25-10372-s001.zip › Supplemental figures 2/original western blot data set2_ds/WBdata2Fig1C-2Aii-ds.tif]

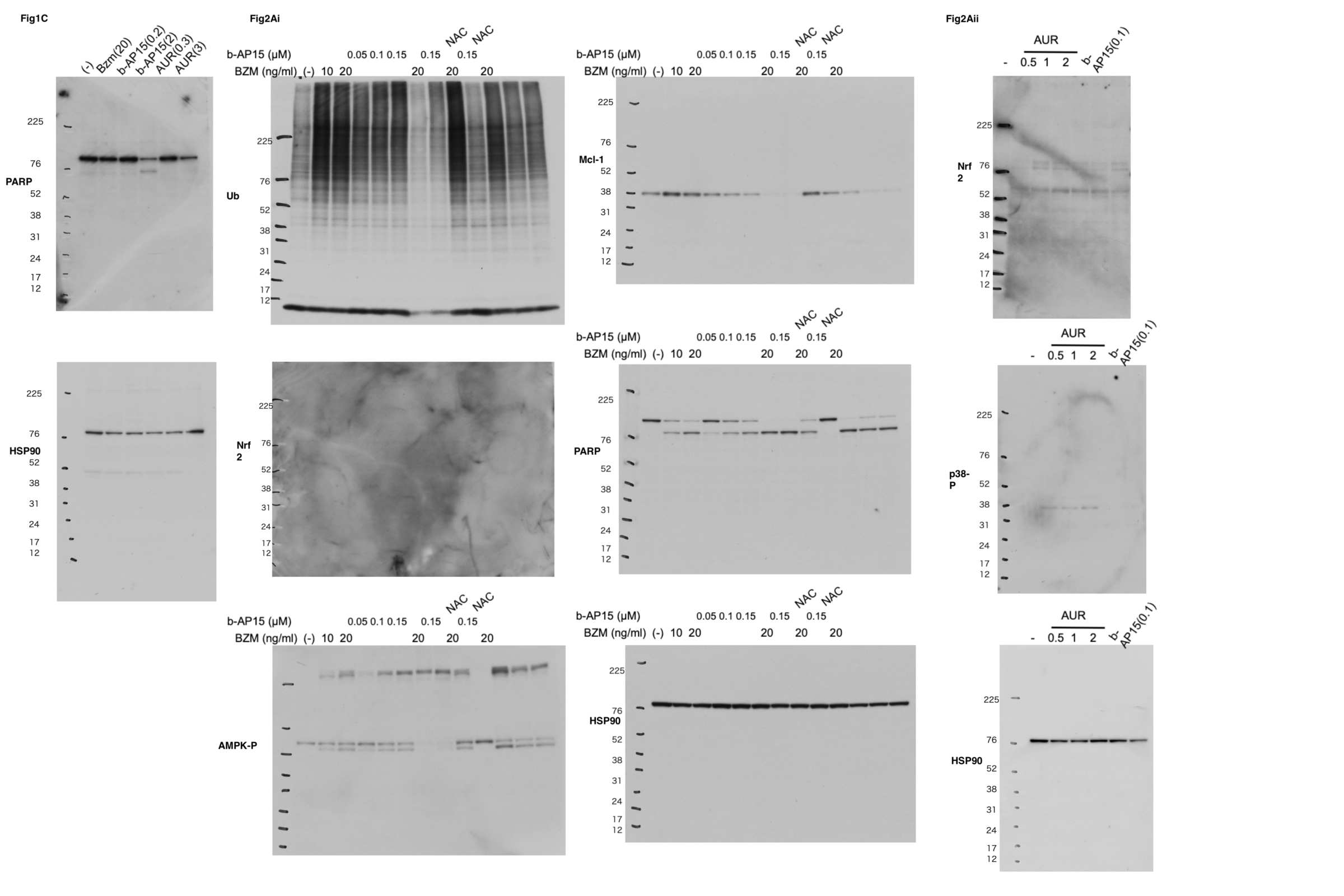

Supplement: Supplementary file 1 [file ijms-25-10372-s001.zip › Supplemental figures 2/original western blot data set2_ds/WBdata2Fig2Aiii-ds.tif]

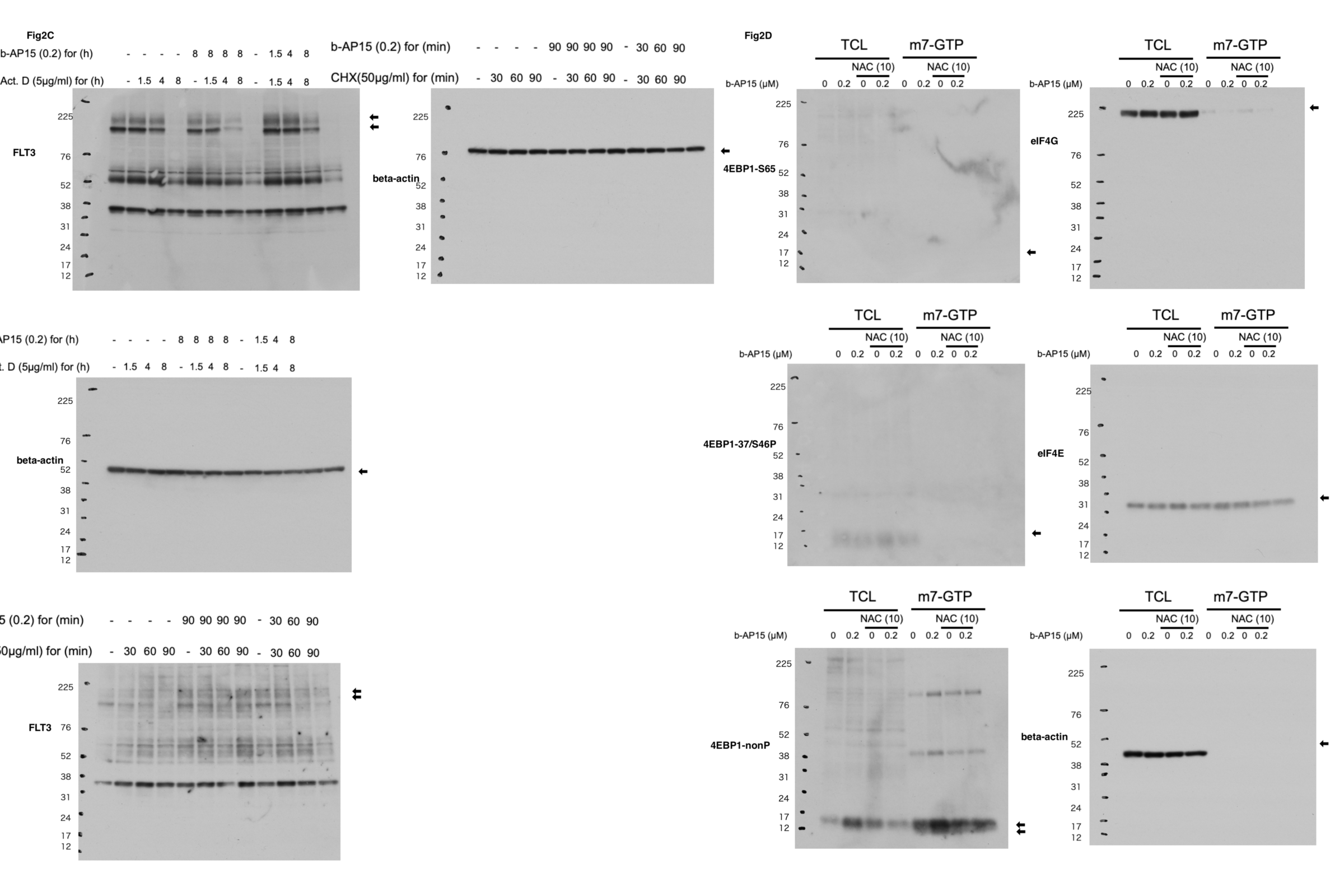

Supplement: Supplementary file 1 [file ijms-25-10372-s001.zip › Supplemental figures 2/original western blot data set2_ds/WBdata2Fig2C2D-ds.tif]

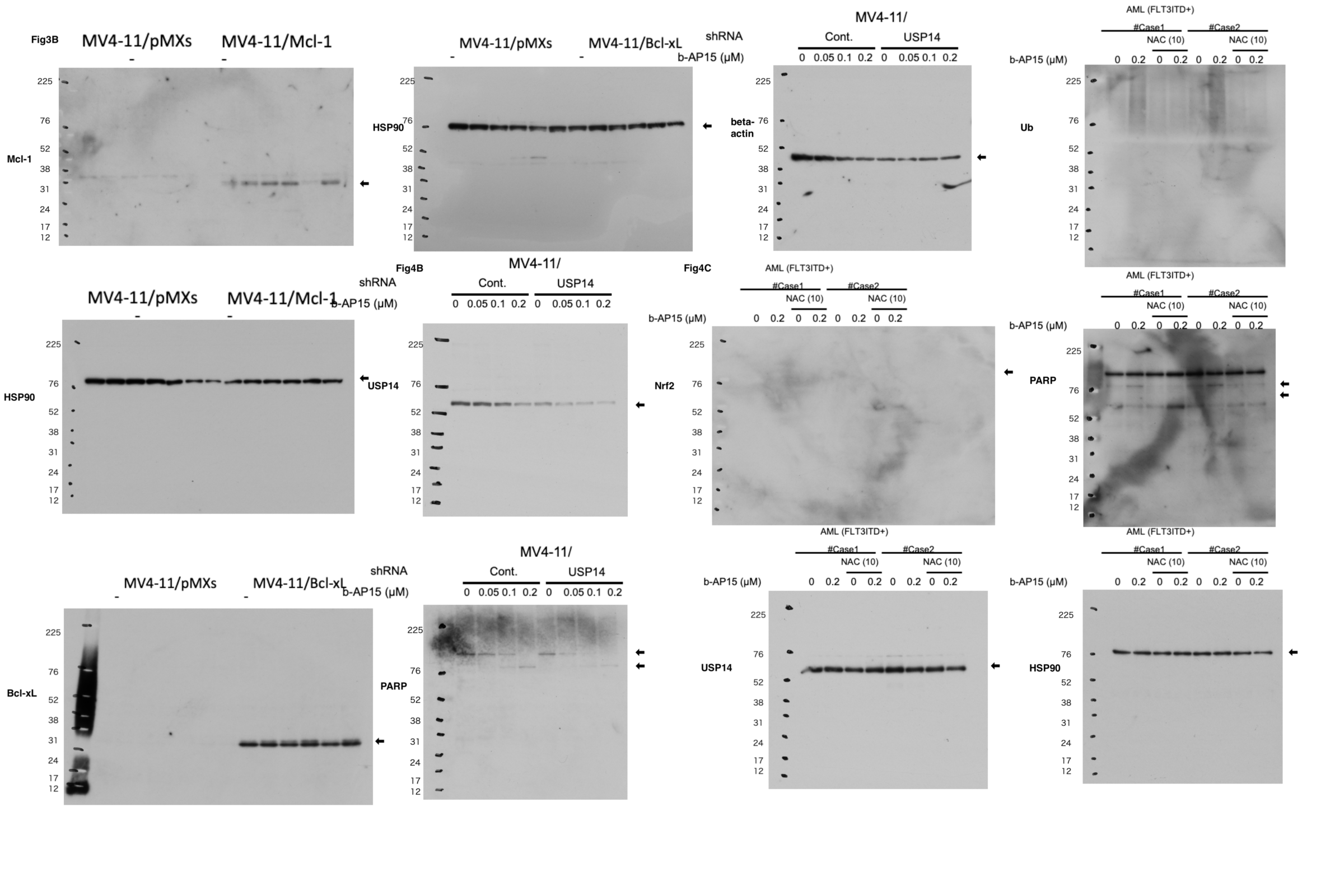

Supplement: Supplementary file 1 [file ijms-25-10372-s001.zip › Supplemental figures 2/original western blot data set2_ds/WBdata2Fig3B-ds.tif]

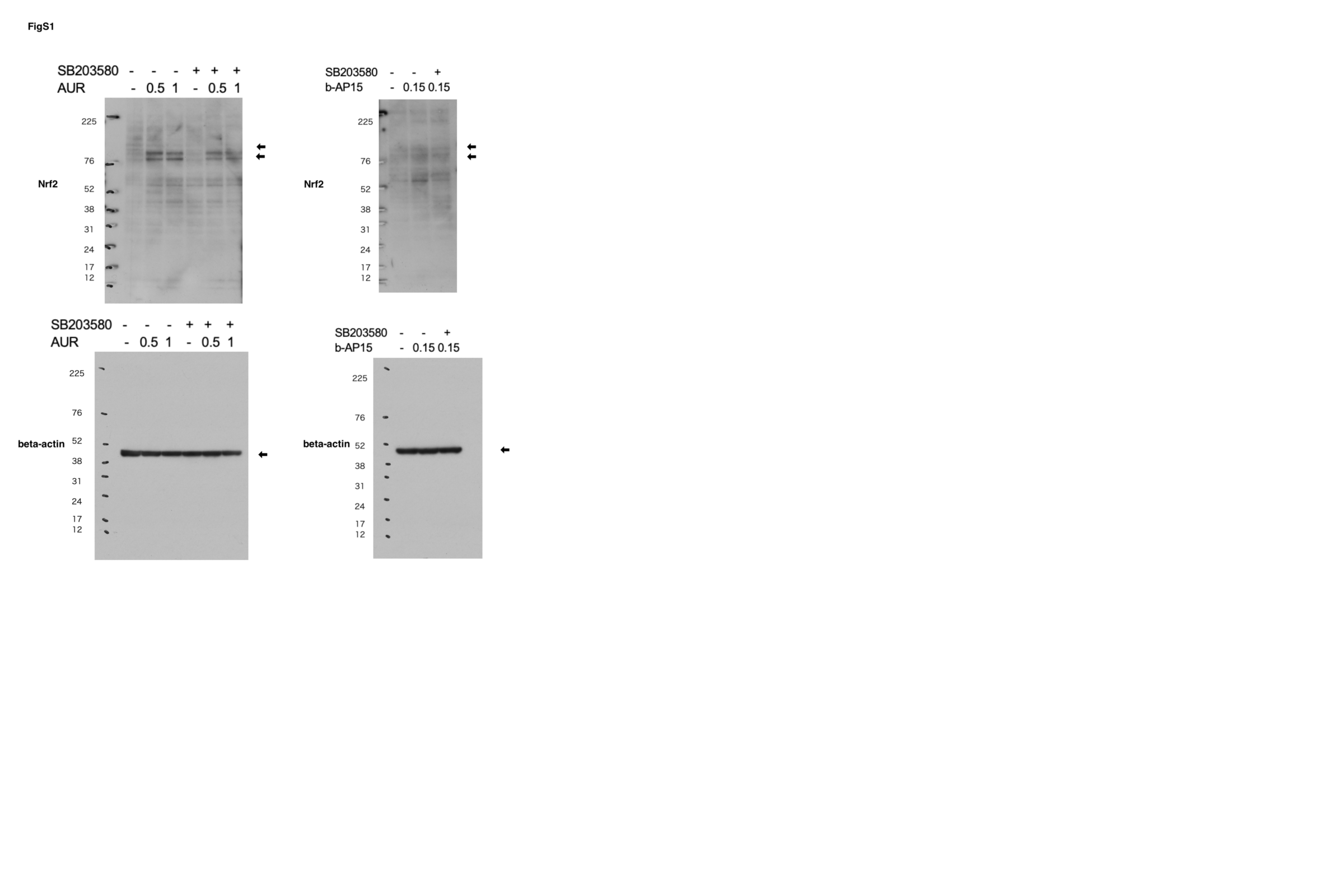

Supplement: Supplementary file 1 [file ijms-25-10372-s001.zip › Supplemental figures 2/original western blot data set2_ds/WBdata2FigS1-ds.tif]
